# Self-Directed Engagement with a Mobile App (Sinasprite) and Its Effects on Confidence in Coping Skills, Depression, and Anxiety: Retrospective Longitudinal Study

**DOI:** 10.2196/mhealth.9612

**Published:** 2018-03-16

**Authors:** Armando Silva Almodovar, Swatee Surve, David Rhys Axon, David Cooper, Milap C Nahata

**Affiliations:** ^1^ Institute of Therapeutic Innovations and Outcomes The College of Pharmacy The Ohio State University Columbus, OH United States; ^2^ Litesprite Bellevue, WA United States; ^3^ College of Pharmacy Department of Pharmacy Practice and Science University of Arizona Tucson, AZ United States; ^4^ Joint Base Lewis-McChord Tacoma, WA United States

**Keywords:** mental health, retrospective studies, longitudinal studies, mobile apps, anxiety, depression

## Abstract

**Background:**

Inadequacies in mental health care coverage remain an enormous problem in the United States. Barriers include scarcity of accessible mental health care professionals. Use of a mental health mobile app incorporating social cognitive theory may help improve confidence in coping skills and improve anxiety and depression. Sinasprite is a mobile app that recruited users via self-referral and clinician referral. Users completed questionnaires to obtain demographic and medical histories. At baseline and 6-week follow-up, users completed the Patient Health Questionnaire 8 (PHQ-8), General Anxiety Disorder 7-Item (GAD-7), and the Coping Self-Efficacy Scale (CSE). It is unknown how self-directed use of a mobile app improves confidence in coping skills and its effects on self-reported depression and anxiety.

**Objective:**

The objective of this study was to evaluate the Sinasprite database to assess self-directed engagement and how use of this mobile app impacted self-reported confidence in coping skills and severity of depression and anxiety.

**Methods:**

This retrospective longitudinal study involved users recruited via clinician referral and self-referral through social media and news media. Questionnaires were used to record demographic, medical, and prescription medication histories. Mental health status was assessed via PHQ-8, GAD-7, and CSE questionnaires. A deidentified dataset reporting mobile app use data was provided to investigators. Individuals with verifiable usage data and at least one completed questionnaire at 6 weeks of use were included. Mann–Whitney *U* and Kruskal-Wallis tests were used to assess whether demographic data and psychotherapy were related to baseline questionnaire scores and usage. A Spearman rho (ρ) test was used to assess the relationship between improvement in the CSE and GAD-7 and PHQ-8 questionnaires. Changes in mental health status were assessed using Wilcoxon signed-rank test. A mixed-effects repeated-measures linear regression model assessed the main effects of time, concomitant counseling, and psychotropic prescription medication use on mental health status.

**Results:**

Thirty-four users were eligible for inclusion in the analysis. Users were predominantly female, white, married, and college educated. At baseline, 35% (12/34) of respondents reported the use of individual/group counseling, and 38% (19/34) reported using prescription medications for their mental health. The median user completed 5.7 (interquartile range 2.7-14.1) trackable activities per week. Statistically significant improvements using a Wilcoxon signed-ranked test were observed in the PHQ-8 (*P*<.001), GAD-7 (*P*=.002), and CSE (*P*<.001) questionnaire scores. A strong positive correlation between improvement in the GAD-7 and CSE questionnaire scores (ρ=.572, *P*=.001, n=28) was observed. The mixed-effects repeated-measures regression model revealed a statistically significant effect of time on improvements in the PHQ-8 (*P*<.001), GAD-7 (*P=*.007), and CSE (*P*=.001).

**Conclusions:**

This 6-week retrospective study showed that self-directed use of the mobile app, Sinasprite, resulted in significant improvements in self-reported questionnaire scores reflecting depression, anxiety, and confidence in coping skills.

## Introduction

Approximately 43 million adults in the United States experienced mental illness in 2015 [1]. The national cost of mental illness was $467 billion in the United States in 2012 [2]. Uncontrolled mental health conditions are associated with increased costs from medications, clinic visits, hospitalizations, incarceration, homelessness, emergency room visits, and premature mortality [3]. The financial burden, stigma, lack of perceived need for treatment, and gaps in access to health care professionals and facilities are among the barriers to attainment of mental health care in the United States [4,5]. Thus, viable alternatives are needed to address this escalating mental health care crisis.

Depression and anxiety are ranked the first and sixth global causes of disability, respectively [6]. Contemporary practice guidelines recommend initiation of antidepressant medications and/or psychotherapy such as cognitive behavioral therapy (CBT) in the treatment and management of patients’ depression and anxiety [7-11]. Furthermore, one-half of all patients with anxiety [6] and one-third of those with depression [12] may benefit from early CBT interventions.

Two-thirds of the American population owns a smartphone, thereby presenting an opportunity for health care services that can overcome geographical and financial limitations [5]. This resulted in the release of 165,000 health-related mobile apps to the public in 2015 on Android and iOS platforms [5]. However, only 7% of these mobile apps provided services to track, assess, or treat mental health conditions [5].

A study by Burns et al demonstrated that a mobile app, which incorporated the use of machine learning, behavioral training, and coaching, was able to assist in the care of patients with major depressive disorder [13]. Lyet et al found the delivery of behavioral activation and mindfulness therapy through a mobile app, complemented by face-to-face therapy, to be an effective means of improving depression severity [14]. The Get Happy Program based on the principles of CBT resulted in improved self-reported Patient Health Questionnaire 9 (PHQ-9) scores up to 3 months after program completion [15]. Another study found a mobile app that incorporated CBT, behavioral activation, mindfulness, and psychoeducation in conjunction with coaching, significantly improved self-reported depression severity among users [16]. Cognitive bias modification interventions for attention via a mobile app were beneficial in the management of anxiety among patients [17]. Arean et al found that individuals with moderate levels of depression may benefit most from mobile apps [18]. Meta-analyses reported that smartphone interventions positively improved symptoms of depression and anxiety among users [19,20]. In addition, a recent systematic review also concluded that the utilization of CBT therapies via a mobile app platform might improve the management of a variety of mental health conditions [21].

Although there are significant published data on the effects of mobile health apps on depression and anxiety, little is known about the effects of these interventions on coping skills and their relationship with the management of depression and anxiety severity. Coping is defined as the behavioral and cognitive efforts individuals use when faced with psychological, emotional, and physical stressors [22,23]. Problem and/or emotional-focused coping strategies may be used to address these stressors [22,23]. Perceived self-efficacy in coping is expected to reflect one’s confidence in their ability to cope with stressors, threats, and challenges [23]. Individuals with higher levels of coping self-efficacy are expected to better mediate potential stressors and challenges [24]. Previous research has highlighted the inverse relationship between coping self-efficacy with depression and anxiety [23-25]. Furthermore, the increased uses of coping strategies have been shown to reduce severity of depression and anxiety [26]. Little is known about how a mobile app designed to improve confidence in coping skills may impact depression and anxiety severity.

Sinasprite is a self-directed mobile app developed using Bandura’s social cognitive theory that includes elements of CBT and mindfulness strategies to improve an individual’s ability to cope with stressors and is expected to decrease the severity of an individual’s anxiety and depression. Litesprite (Bellevue, WA, USA), an organization that develops mental health mobile apps, released Sinasprite in the iOS and Android app stores as a beta (Multimedia Appendix 1). To download the mobile app, users accessed the Litesprite website, completed voluntary questionnaires, and received a beta key permitting access to the mobile app. Users were recommended to use Sinasprite for 6 weeks and to complete a voluntary follow-up questionnaire afterward. The objective of this study was to conduct an assessment of the Litesprite database to evaluate engagement with the mobile app and how a self-directed mobile app can impact confidence in coping skills and depression and anxiety severity.

## Methods

This retrospective longitudinal study evaluated user engagement and outcomes associated with the use of Sinasprite, a native mobile app developed by Litesprite. Investigators received a deidentified dataset from the mobile app development team containing usage data, questionnaire responses, and demographic data. The Ohio State University Institutional Review Board deemed this study to be exempt from human subject’s research.

### Mobile App Design

This mobile app was developed using Bandura’s social cognitive theory and included elements of CBT and mindfulness-based stress reduction [27]. The game-based modules incorporate features such as visualization, diaphragmatic breathing, meditation, anxiety journal writing, augmented reality exercise, and mindfulness. Users participate in these modules to help Socks the Fox (a digital avatar) become a Zen master. The mobile app also uses intrinsic incentives and in-game prompts and rewards to reinforce engagement and the use of multiple modules. Repeated use of the mobile app is expected to gradually improve an individual’s self-efficacy, sense of self-control, reinforcements, and coping skills to ultimately improve the management of their stress, depression, and anxiety. Sinasprite is a native mobile app for iOS and Android operating systems that does not require an active internet connection. Once the mobile app is finalized and updated, prospective users would be able to access it through the iOS and Android mobile app stores.

### Recruitment

The mobile app was released to the public in a live beta to allow users to use and potentially benefit from it with minimal intervention and support from health care practitioners. Anyone aged 18 years or older was invited to use the mobile app. Users were recruited via clinician referrals and self-referral through social media and news media such as Facebook advertisements and presentations and news articles published in *VentureBeat*, *Puget Sound Business Journal*, *The Northwest Guardian*, *GeekWire*, *Elevar*, *Serious Games Market*, *SVP Fast Pitch*, *The Huffington Post*, *Casual Connect*, *Seattle Met*, *Counseling Washington*, *Medgadget*, *Chase*, *425 Business*, *The Law of Startups*, *Seattle Health Innovators*, *Marketplace*, *Women 2.0*, *The Seattle Times*, *Portland Business Journal*, *International Business Times*, and *iMedicalApps*. Potential users were directed to the Litesprite website and signed up to use the mobile app. Users were then emailed a link to a secure website where they completed a voluntary questionnaire. On submission, users were sent a beta key via email within a day, providing access to the beta version of the mobile app. They subsequently downloaded the mobile app from the app store of choice and used the beta key to access the mobile app.

### Questionnaires

Users were electronically sent access to a secure website where they completed a voluntary questionnaire requesting demographic information, medical history, use of psychotherapy and prescription medications, and mental health status via the Patient Health Questionnaire 8 (PHQ-8) [28,29], General Anxiety Disorder 7-Item (GAD-7) [30], and the Coping Self Efficacy Scale (CSE) questionnaires [23]. The PHQ-8 was used over the PHQ-9 to measure depression severity because the nonproctored format of the survey prevented the appropriate assessment of suicidal ideation, which is uncommon in the general population [29]. The GAD-7 and CSE questionnaires were used to assess anxiety severity and confidence in coping skills [23,30]. Users did not need to completely fill out the survey to be able to submit. After 6 weeks of using the mobile app, users were sent a link to a secure website, where they were able to complete the mental health status questionnaires (PHQ-8, GAD-7, and CSE). Questionnaires with incomplete PHQ-8 or GAD-7 questions were excluded from analysis. CSE questionnaires were included in the analysis if at least 80% (21/26) of the questions were answered [23]. Missing responses were replaced by the mean of completed items, resulting in a corrected sum [23].

### Data Analysis

Data were organized and coded in IBM SPSS Statistics (v24.0; Armonk, NY, USA) and were assessed for normality using the Wilk-Shapiro test and histograms. All users were included in the initial dataset. Users who did not complete any of the surveys after 6 weeks of use or did not have verifiable usage data were excluded. Due to the low sample size and non-normal distribution of the data, Mann-Whitney *U* and Kruskal-Wallis statistics were used to assess the relationship between demographic data, psychotherapy, baseline PHQ-8, GAD-7, or CSE questionnaire scores and the usage metrics. A 2-tailed a priori alpha level of .05 was used.

### Mobile App Engagement

Several indicators were used to evaluate the mobile app. Included were the average length of in-game session, completed meditation sessions, mindfulness paintings, anxiety journal entries, and self-assessment questions. Given the in-development (beta) status of the mobile app, it was not possible to collect data from some modules. These included the fishing module and an augmented reality exercise module that encouraged walking. The weekly amount of user activity was calculated by adding the number of completed activities and dividing by 6 weeks. Although the mobile app was intended to be used several times a week, users were encouraged to use the modules in the frequency they felt will be of most benefit to them. This was intended to allow users to determine their own experience and make use of the app as “nonconfrontational” as possible [31]. Thus, adherence could not be adequately assessed in the scope of this study [31]. The Sinasprite development team also provided investigators with a retention rate of users included in the analysis.

### Relationships Between Outcome Measures

A Spearman rho (ρ) test was used to assess the relationship between improvement in the CSE and GAD-7 and PHQ-8 results. A preliminary analysis using a scatter plot of the results was performed to ensure the relationship between improvements in the questionnaire scores followed a monotonic relationship.

### Effects of Sinasprite on Self-Reported Questionnaire Outcomes

To assess the change in self-reported questionnaire outcomes among users, a Wilcoxon signed-rank test was performed. This test was then repeated with users who reported no concomitant therapies. Cohen *d* effect size was calculated for each test [32].

### Mixed-Effects Repeated-Measures Linear Regression Model

To further evaluate the effects of using the mobile app on mental health, a mixed-effects repeated-measures linear regression model using unstructured variance, restricted maximum likelihood, and intercepts was conducted. This method was chosen because of its superiority to analysis of variance in assessing correlations [13]. Q-Q plots and a Wilk-Shapiro test were used to assess the appropriateness of the model for each mental health status outcome. Transformations were used to normalize data when required. The main effects of time, receipt of prescription drug therapy, and individual or group counseling were included in the linear regression model.

## Results

**Figure 1:**
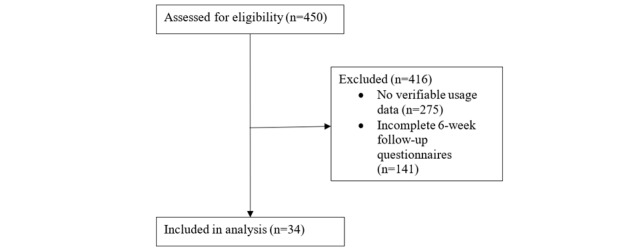
Consolidated Standards of Reporting Trials (CONSORT) flow diagram illustrating exclusion criteria.

The sample included data from 450 users of the mobile app. However, 275 users were excluded because of lack of verifiable usage data, and an additional 141 were excluded for not completing at least one 6-week follow-up questionnaire (PHQ-8, GAD-7, or CSE). The final sample for the analysis included 34 users (Figure 1).

In this study, users included for analysis were predominantly female 77% (26/34), white 41% (14/34), married 62% (21/34), and college educated 71% (24/34) with a median age of 40 (interquartile range [IQR] 32.75-50.75) years. Moreover, 35% (12/34) of users reported receiving individual or group counseling, and 38% (13/34) reported using prescription medications for their mental health (Table 1). After 6 weeks of use, 74% (25/34) of users who were included in the analysis continued to use the mobile app. Retention of users who were excluded was unable to be assessed. Users who reported attending individual or group counseling sessions were more likely to report a higher GAD-7 (*P*=.04) than those who did not receive counseling. Individuals currently using prescription medications for their mental health conditions were more likely to report higher baseline GAD-7 (*P*=.01) and PHQ-8 (*P*=.03) and lower CSE (*P=*.01) questionnaire scores compared with their counterparts. Use of the mobile app was not significantly associated with demographic characteristics or receipt of prescriptions or counseling services for their mental health conditions.

### Mobile App Engagement

Mobile app usage data are presented in Table 2. The median user averaged 6 min per session and used the mobile app once a week. The most used feature was self-assessment questions with users completing a median of 15 questions. The second most used feature was meditation; a median of 5.5 sessions was completed, and users meditated for 1 to 3 min. Completion of paintings and use of anxiety journal entries were comparable with a median of 4 and 3.5 per user, respectively. Users performed a median of 5.7 trackable activities per week. However, one user completed the pre- and postquestionnaires and yet completed no activities in the first 6 weeks, whereas the top 10 most active users completed 12 to 50 activities per week. These data highlight the large degree of interuser variability with the use of the mobile app. There was a moderate to strong, positive correlation between journal entries and self-assessment questions (ρ=.418, n=34, *P*=.001), meditation sessions (ρ=.631, n=34, *P*<.001), and paintings (ρ=.681, n=34, *P*<.001). A strong positive correlation between meditation sessions and paintings (ρ=.927, n=34, *P*<.001) also was observed. Use of the mobile app was moderately and positively correlated with the baseline self-reported GAD-7 questionnaire score (ρ=.365, n=32, *P*=.04).

**Table 1:** Demographic and clinical characteristics of users of Sinasprite mobile app (N=34). Percentages may not equal to 100% because of rounding.

Characteristics		Statistics
Age in years, median (interquartile range)		40 (33-51)
**Gender, n (%)**		
	Male	6 (18)
	Female	26 (77)
	Prefer not to say	2 (6)
**Highest level of education, n (%)**		
	Did not graduate high school	1 (3)
	High school diploma	9 (27)
	Bachelor’s degree	10 (30)
	Graduate degree	14 (41)
**Racial background, n (%)**		
	White	14 (41)
	Asian	1 (3)
	Prefer not to say	19 (56)
**Marital status, n (%)**		
	Single or never married	6 (18)
	Married or partnered	21 (62)
	Separated	2 (6)
	Divorced	3 (9)
**Household income before taxes in US dollars, n (%)**		
	$20,000-$29,999	3 (9)
	$30,000-$49,999	2 (6)
	$50,000-$69,999	1 (3)
	$70,000-$99,999	2 (6)
	$100,000-$149,999	2 (6)
	>$150,000	2 (6)
	Prefer not to say	22 (65)
**Received individual or group counseling, n (%)**		
	Yes	12 (35)
	No	21 (62)
	Did not say	1 (3)
**Used prescription medication for behavioral health, n (%)**		
	Yes	13 (38)
	No	19 (56)
	Did not say	2 (6)

**Table 2:** Frequency of Sinasprite mobile app use by users.

Usage data per user	Median (interquartile range)
Self-assessment questions completed	15.0 (5.0-41.3)
Number of meditation sessions completed	5.5 (2.0-13.3)
Mindfulness paintings completed	4.0 (1.0-12.0)
Anxiety journal entries completed	3.5 (1.75-8.3)
Sinasprite activities per week	5.7 (2.7-14.1)
Sinasprite total activity	34.0 (16.0-84.5)
Total number of sessions	6.0 (3.0-13.85)
Average length of session (min)	6.0 (3.8-8.5)

### Relationships Between Outcome Measures

The relationship between improvement in the CSE questionnaire and the GAD-7 and PHQ-8 questionnaire scores was assessed. Preliminary analysis indicated no violation in the assumption monotonicity. There was a strong positive correlation between improvement in the GAD-7 and CSE questionnaire scores (ρ=.572, *P*=.001, n=28). However, there was a statistically insignificant, small, positive correlation observed between improvement in the PHQ-8 and the CSE questionnaire scores (ρ=.178, *P*=.37, n=28).

### Effects of Sinasprite on Self-Reported Questionnaire Outcomes

**Figure 2:**
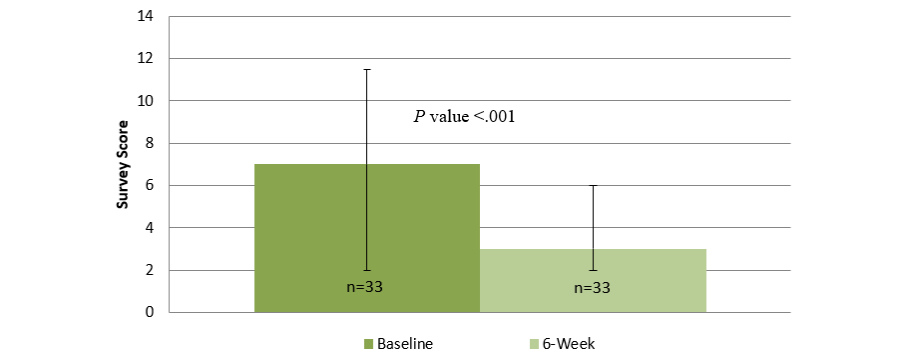
Changes in self-reported depression at baseline and after 6 weeks of using the Sinasprite mobile app on the Patient Health Questionnaire 8 (PHQ-8).

**Figure 3:**
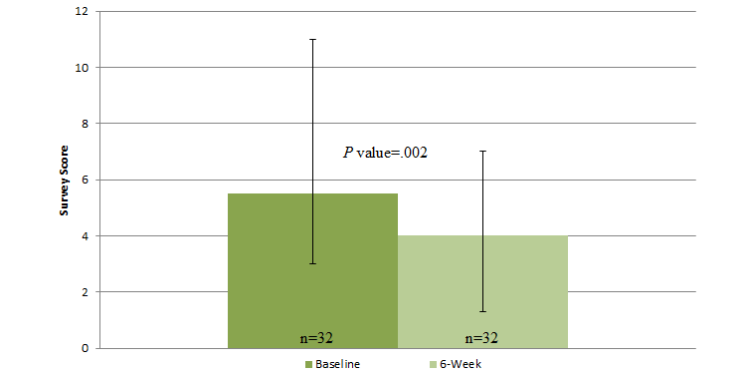
Changes in self-reported anxiety at baseline and after 6 weeks of using the Sinasprite mobile app on the General Anxiety Disorder 7-Item (GAD-7).

**Figure 4:**
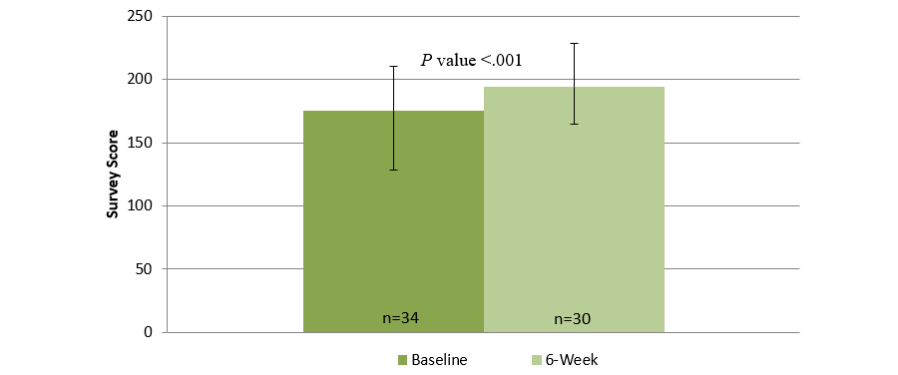
Changes in self-reported confidence in coping skills at baseline and after 6 weeks of using the Sinasprite mobile app on the Coping Self-Efficacy Scale (CSE).

Before using the mobile app, the median user reported a PHQ-8 score of 7 (IQR=2.0-11.5, indicating mild depression) and a GAD-7 score of 5.5 (IQR=3.0-11.0, indicating mild anxiety) [29,30]. After 6 weeks of using the mobile app, the median user reported a PHQ-8 score of 3.0 (IQR=2.0-6.0, indicating none or minimal depression) and a GAD-7 score of 4.0 (IQR=1.3-7.0, indicating no or minimal anxiety symptoms) [29,30]. Figures 2-4 illustrate changes in the PHQ-8, CSE, and GAD-7 self-reported questionnaire scores. The CSE at baseline was 174.9 (IQR=128.0-210.6), which improved to 194.0 (IQR=164.3-228.5) after 6 weeks of using the mobile app. A statistically significant improvement was observed in the PHQ-8 (n=32, *z*=−3.501, *P*<.001, effect size=0.44), GAD-7 (n=31, *z*=−3.138, *P*=.002, effect size=0.40), and CSE (n=30, *z*=−3.557, *P* ≤.001, effect size=0.46) questionnaire scores after 6 weeks of engagement with the mobile app.

Before using the mobile app, the median user not currently receiving psychotropic medications or counseling services reported a PHQ-8 of 4.5 (IQR=1.3-10.5, indicating mild depression) and a GAD-7 score of 3.0 (IQR=2.0-9.0, indicating none or minimal anxiety) [29,30]. After 6 weeks of using the mobile app, the median user reported a PHQ-8 score of 2.0 (IQR=1.0-4.0, indicating none or minimal depression) and a GAD-7 score of 3.5 (IQR=0.3-4.8, indicating none or minimal anxiety) [29,30]. The CSE at baseline was 178.0 (IQR=161.0-215.5), which improved to 219.0 (IQR=189.0-230.0) after 6 weeks of using the mobile app. Significant improvements were observed in the PHQ-8 (n=16, *z*=−2.884, *P*=.004, effect size=0.51), GAD-7 (n=15, *z*=−2.282, *P*=.02, effect size=0.42), and CSE (n=15, *z*=−2.840, *P*=.005, effect size=0.52) questionnaire scores, after 6 weeks of engagement with the mobile app.

### Mixed-Effects Repeated-Measures Linear Regression Model

To assess the effects of concomitant therapies on outcomes, the changes in the self-reported questionnaire outcomes (PHQ8, GAD-7, and CSE) were modeled using a mixed-effects repeated-measures linear regression model. The variables included main effects of time, receipt of individual or group counseling, and use of prescription medications. The GAD-7 scores were transformed using square root arithmetic. Wilk-Shapiro test and Q-Q plots indicated a normal distribution, and no further transformations were necessary. This model assessed changes before and after 6 weeks of using the mobile app. The complete results can be found in Table 3. There was a significant effect of time on the PHQ-8 (*P*<.001), GAD-7 (*P*=.007), and CSE (*P*<.001) questionnaire scores. In addition, a significant effect of prescription medications was noted on the PHQ-8 (*P*=.003) and CSE (*P*=.009); however, statistical significance was not achieved for the GAD-7 survey. The main effect of counseling or group therapy was not statistically significant for any of the questionnaires. The main effect of time indicated that there was a statistically significant improvement between the pre- and posttests for all 3 questionnaires after controlling for concomitant therapies. Use of prescription medications was also a statistically significant variable for depression severity (PHQ-8) and confidence in coping skills (CSE).

**Table 3:** Results from mixed-effects repeated-measures linear regression model.

Questionnaires		F statistic (degrees of freedom)	*P* value
**Patient Health Questionnaire (PHQ-8)**			
	Intercept	67.1 (1.0,30.0)	<.001^a^
	Time	15.5 (1.0,30.0)	<.001^a^
	Individual or group counseling	0.1 (1.0,30.0)	.78
	Prescription medication use	10.2 (1.0,31.0)	.003^a^
**General Anxiety Disorder 7-Item (GAD-7)**			
	Intercept	186.9 (1.0,28.0)	<.001^a^
	Time	8.4 (1.0,29.0)	.007^a^
	Individual or group counseling	1.7 (1.0,29.0)	.21
	Prescription medication use	3.8 (1.0,29.0)	.06
**Coping Skills Self-Efficacy Survey (CSE)**			
	Intercept	731.9 (1.0,30.0)	<.001^a^
	Time	17.7 (1.0,29.0)	<.001^a^
	Individual or group counseling	0.3 (1.0,28.0)	.56
	Prescription medication use	7.9 (1.0,28.0)	.009^a^

^a^Significant at the .05 significance level.

## Discussion

### Comparison With Prior Work

Over 4800 areas around the United States reported a shortage of mental health providers in 2017 [33]. Furthermore, one-half of patients with mental illness reported not to have received any mental health care in 2014 [34]. Use of mobile apps may be able to reach individuals beyond geographical, financial, and social circumstances. The results of our study are in line with previous meta-analyses that concluded mobile app–based interventions might positively impact the severity of depression and anxiety [19,20].

The results from this study are similar to previously published studies that detailed the positive effects of different mobile app interventions on depression severity [14-16,18,35-37]. It is important to note that several of these studies included access to additional resources or health care practitioners [14,15,35,36]. Furthermore, the effect size of the present mobile app (effect size=0.44) on depression severity is comparable with other self-guided mobile apps (effect size=0.21-0.70) [35]. Regarding anxiety severity, the results from this study are similar to others that demonstrated that mobile apps may positively improve management of anxiety [17,36-39]. However, it is difficult to adequately compare the results of this study with others because of the differences in populations, methodology in assessing mental health status, execution of interventions, and follow-up period. This study also found the mobile app positively impacted self-confidence in coping skills. To our knowledge, this is the first study to assess how a mobile app may influence confidence in coping skills.

### Principal Findings

Key findings from this study showed self-directed engagement of Sinasprite after 6 weeks of use was associated with statistically significant improvements in self-reported PHQ-8, GAD-7, and CSE questionnaire scores. Statistically significant improvements were seen even after controlling for concomitant prescription medication and counseling or group therapies. This study also found a strong positive correlation between improvement in the CSE and GAD-7 questionnaire scores. Unexpectedly, a statistically insignificant correlation was detected between improvement in the CSE and the PHQ-8 questionnaires.

Over the 6-week study period, the median user performed approximately 6 activities per week in the Sinasprite app. Although users were encouraged to use the mobile app to meet their needs, not all activities were tracked and assessed; thus, an underestimation of actual engagement in the mobile app may have occurred. Moreover, the beta status of the mobile app may have resulted in lower levels of user engagement; use of the mobile app may substantially increase when a fully functional version is made available to the general public. However, it is noteworthy that the degree of engagement shown from the beta version resulted in statistically significant improvements in questionnaire scores for depression and anxiety severity and coping skills. Summary data provided by the Sinasprite development team reported that 74% (25/34) of users included in the analysis continued to use the app after 6 weeks of use. This is consistent with previously reported retention rates, ranging from 10% to 70%, for internet and mobile app interventions [16]. However, it is important to note, among the entire sample, only 38.9% (175/450) of users actually presented with usage data.

Use of the Sinasprite mobile app for various features may subsequently prompt use of other features. For example, the mobile app prompted users to make a painting after completion of a meditation session to promote self-awareness from the meditation experience. Also, on completion of an anxiety journal, entry users were prompted to complete a breathing exercise although this is an untracked feature. The moderate to strong correlations detected between the trackable features suggest that users were not selectively using specific features and were likely to use multiple features that may be guided by in-game prompts. Further study is needed to assess which features are more valuable for particular populations once the mobile app is finalized and released to the public.

An underestimation of the effects of the mobile app on change in self-reported questionnaires responses may have occurred for several reasons. Users may have experienced glitches and errors in the beta version that impeded their ability to benefit from the mobile app fully. Despite this limitation, it is important to emphasize that a statistically significant change was still observed between the initial and follow-up questionnaire scores.

The CSE questionnaire measures one’s confidence in their ability to carry out coping strategies when faced with external stressors and does not have thresholds to differentiate between specific levels of coping [23]. A statistically significant median improvement of 19 points on the CSE questionnaire items suggests that use of the mobile app helped users improve their confidence in the execution of coping strategies. This study also found a strong relationship between improvement in the CSE and GAD-7 scores. These results support the notion that as users improve their confidence in coping skills, they may also improve their ability to handle stressors and anxiety. This is supported by previous research that found coping strategy interventions improved depression and anxiety symptoms [26]. However, the lack of statistical significance between the CSE and PHQ-8 questionnaires conflicted with previous findings and suggested that one’s confidence in their coping skills may not significantly impact depression severity among patients. This finding may have occurred because of the low sample size. It is still important to note that significant improvement in the PHQ-8 survey was observed.

### Limitations

These study results are subject to several limitations. The retrospective nature of this study prevented investigators from accounting for initiation and discontinuation of mental health services during the 6 weeks of the Sinasprite mobile app use and recruitment of specific populations. The study also did not have a control group for comparison. The in-development status of the Sinasprite mobile app may have limited full user engagement. The fact that the vast majority of the original sample was excluded 92.4% (416/450) because of the lack of verifiable usage 61.1% (275/450) or completion of the postuse survey scores 31.3% (141/450) is concerning and leads to greater potential for bias (eg, nonrandom, self-selection). This, however, is expected as dropout rates among internet-based studies can fluctuate between 50% and 90% [18,40]. Comorbidities were not assessed in this study. Length of previous or current therapy was not collected. The unassisted platform of the survey may have resulted in incorrect input by users. Use of the mobile app was not supplemented by administrative or clinical assistance, which may have resulted in lower engagement [16,41,42]. The significant dropout rate in this study is concerning; however, it is important to note that the mobile app assessed is in a beta phase and is still being optimized to improve the user experience. Once a finalized product is made available via iOS and Android platforms, further study is needed to understand how the mobile app is used and how it may impact larger and more diverse populations without the use of additional health care resources.

### Conclusions

This study found that individuals’ scores for self-reported coping skills and depression and anxiety symptoms improved without additional investment of health care resources after 6 weeks of using the Sinasprite mobile app. Although encouraging, further study is warranted to evaluate the fully functional Sinasprite mobile app among a larger and more diverse population with mental health conditions, including anxiety and depression.

## Appendix

Multimedia Appendix 1 Screenshots of the Sinasprite mobile application home screen and two modules.

## Abbreviations

CBT: cognitive behavioral therapy
CSE: Coping Self-Efficacy Scale
GAD-7: General Anxiety Disorder 7-Item
IQR: interquartile range
PHQ-8: Patient Health Questionnaire 8
PHQ-9: Patient Health Questionnaire 9
