# Supplementary material for: Self-Directed Engagement with a Mobile App (Sinasprite) and Its Effects on Confidence in Coping Skills, Depression, and Anxiety: Retrospective Longitudinal Study
Source: JMIR Mhealth Uhealth. 2018 Mar 16;6(3):e64. doi: 10.2196/mhealth.9612 (PMC5878360; doi:10.2196/mhealth.9612)
Supplement: Multimedia Appendix 1 [file mhealth_v6i3e64_app1.pdf]

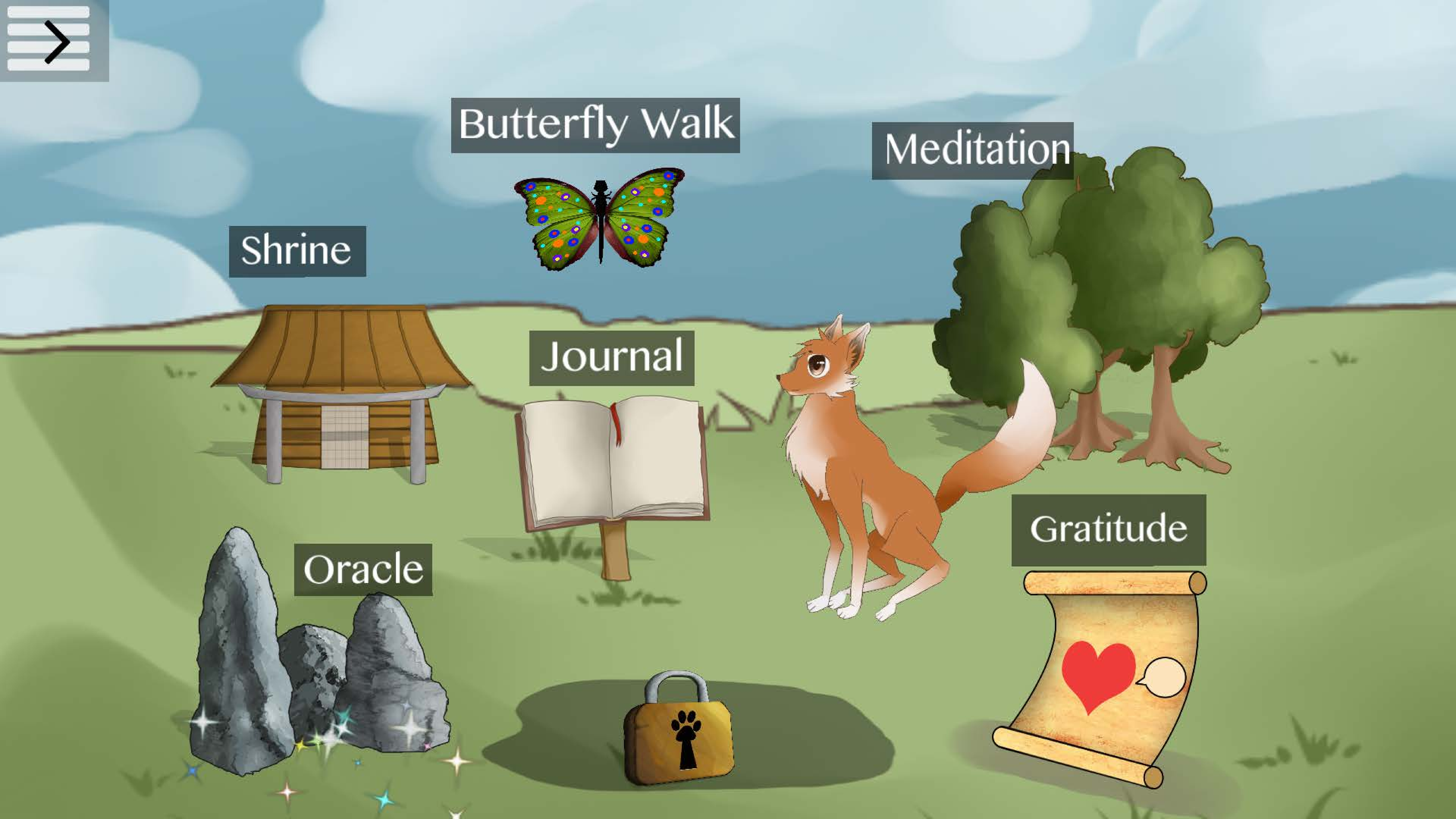
Butterfly Walk
Meditation
Shrine
Journal
Gratitude
Oracle

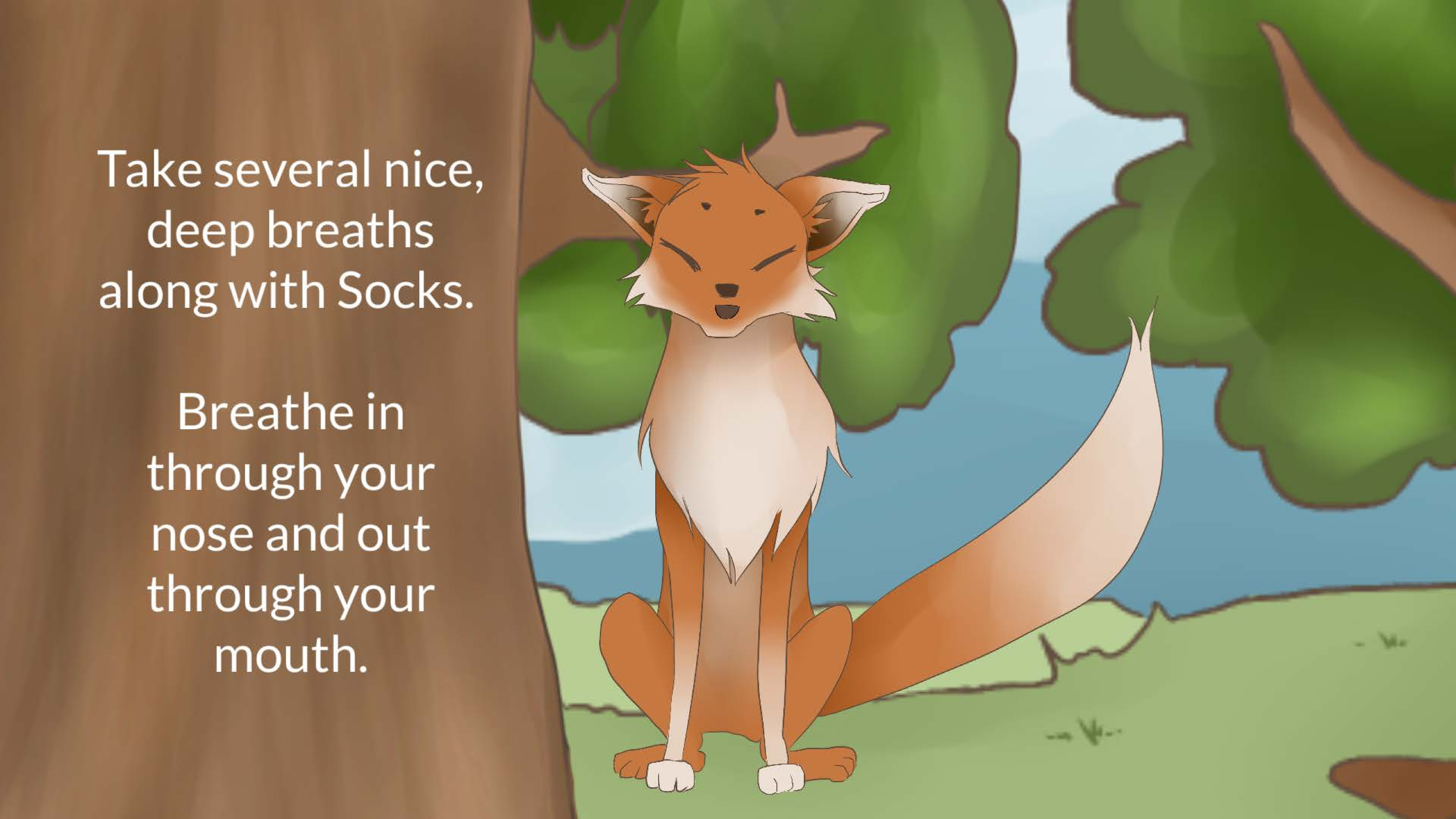
Take several nice, deep breaths along with Socks.
Breathe in through your nose and out through your mouth.

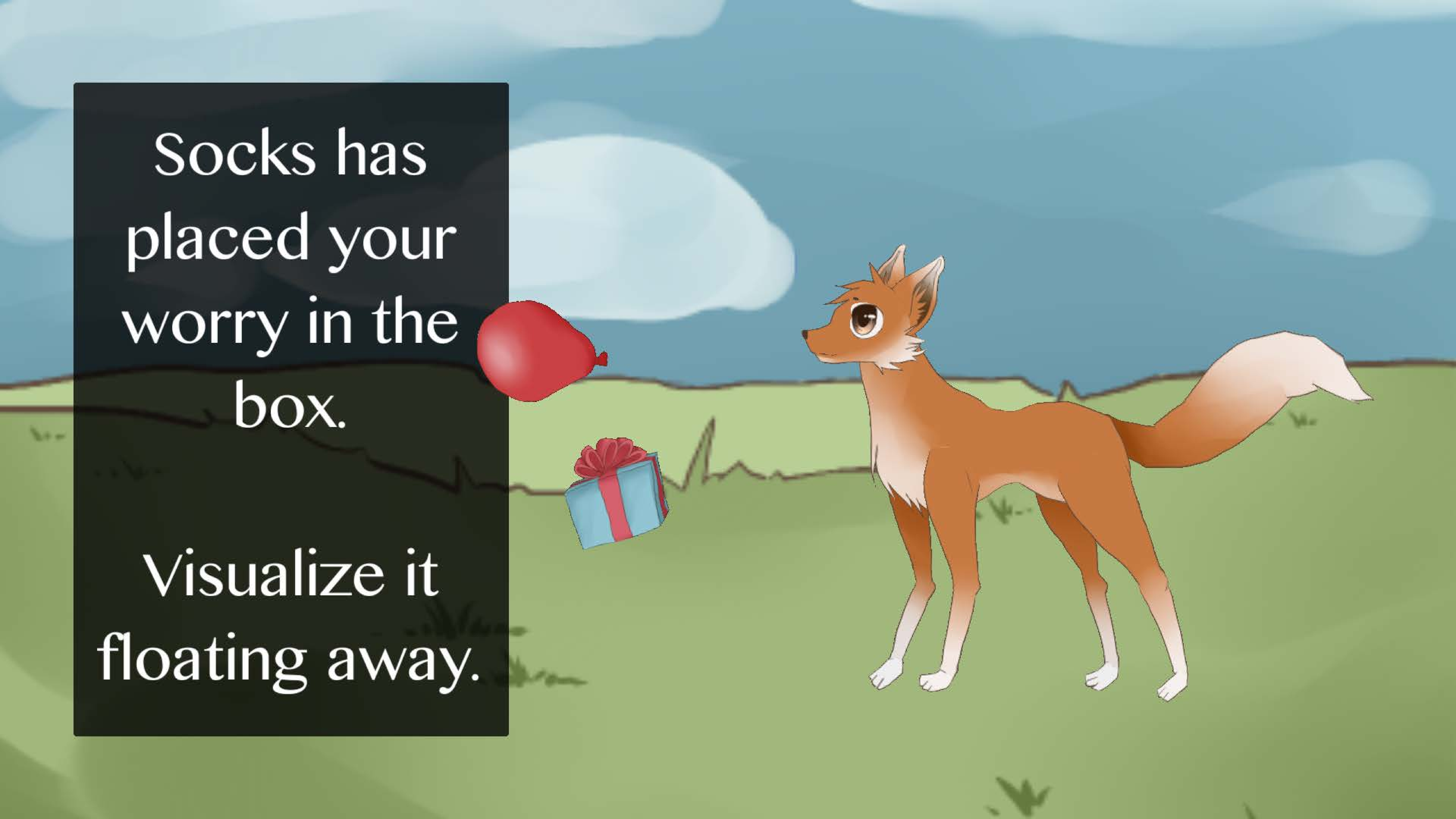
Socks has placed your worry in the box.
Visualize it floating away.
